# Knowledge, attitude, and perceptions about polycystic ovarian syndrome, and its determinants among Pakistani undergraduate students

**DOI:** 10.1371/journal.pone.0285284

**Published:** 2023-05-25

**Authors:** Mehwish Rizvi, Md. Ashraful Islam, Muhammad Tariq Aftab, Atta Abbas Naqvi, Amnah Jahangir, Azfar Athar Ishaqui, Muhammad Zahid Iqbal, Muhammad Shahid Iqbal

**Affiliations:** 1 Dow College of Pharmacy, Dow University of Health Sciences, Karachi, Pakistan; 2 Department of Pharmacy Practice, College of Clinical Pharmacy, Imam Abdulrahman Bin Faisal University, Dammam, Saudi Arabia; 3 Department of Pharmacology, Islam Medical and Dental College, Sialkot, Pakistan; 4 School of Pharmacy, University of Reading, Whiteknights Campus, Reading, United Kingdom; 5 Department of Pharmacy, Tabba Heart Institute, Karachi, Pakistan; 6 Department of Pharmacy, Iqra University, Karachi, Pakistan; 7 Department of Pharmacy Practice, Lahore Pharmacy College, University of Health Sciences, Lahore, Pakistan; 8 Department of Clinical Pharmacy, College of Pharmacy, Prince Sattam Bin Abdul Aziz University, Alkharj, Saudi Arabia; Al-Jouf University College of Pharmacy, SAUDI ARABIA

## Abstract

**Objective:**

The aim of the study was to evaluate knowledge, attitude, perception, and assess the determinants of polycystic ovarian syndrome (PCOS) among undergraduate students.

**Methods:**

A cross-sectional study was conducted among female undergraduate students in Pakistan using a survey. The questionnaire was formulated in English language by a review of literature and expert consensus. The sampling approach was convenient, and survey was available as electronic as well as hardcopy. Data were analyzed using IBM SPSS v23. Descriptive statistics namely mean ($\bar{x}$), standard deviation (SD), or median ($\bar{x}$) and interquartile range (IQR) were used dependent upon data distribution. In addition, range (R) was also utilized to express the results. The logistic and linear regression analyses were also conducted. Study received ethical clearance from ethics committees.

**Results:**

A total of 646 responses were analyzed. The average PCOS knowledge score was 11.58 ± 4.99 (overall), 12.02 ± 4.73 (medical students), 9.36 ± 5.65 (non-medical students) (α = 0.861). 68.6% participants did not feel embarrassed while discussing PCOS in the society, but 67.3% never discussed it with a doctor. Lack of self-knowledge (31.6%) and shyness/reluctance (21.4%) were identified as barriers by most students. Further, obesity, irregular menstrual periods, family history, hirsutism and contraceptive use were observed to be determinants for having PCOS (AOR > 2, p<0.05). The disease knowledge score was higher for participants studying in medical college (β = 0.184), having menstrual periods < 5 days (β = 0.125), and with a family history (β = 0.121) (p<0.05).

**Conclusion:**

The disease knowledge among undergraduates was inadequate. However, there is greater acknowledgement of PCOS as a problem for Pakistani women and barriers have been identified in the study. Conducting awareness campaigns within academic institutions which include promoting disease education, arranging talks, distributing merchandise with disease awareness signage, would greatly help in raising awareness of the disease and lowering stigma and hesitancy.

## Introduction

One of the most common endocrine problems among women of reproductive age is polycystic ovarian syndrome (PCOS) [1–3]. It is characterized by hirsutism, menstrual irregularities, obesity, acne, and alopecia [4]. A prevalence from 5% up to 20% is mentioned in the literature [4–6]. This not only affects the fertility of the women but predisposes them to the risk to diabetes mellitus and depression as well [7, 8]. Available evidence highlights that poor awareness of PCOS may lead to negative outcomes for women [9]. Usually, hormonal profiling and pelvic ultrasound is done to confirm the diagnosis of PCOS [10].

The diagnosis of PCOS is based on several criteria such as irregularities in menstrual cycle, having no periods, etc., excessive androgens or its signs for instance hirsutism, and having cysts in the ovaries [11]. According to the National Health Service (NHS), the disorder can be managed with changing lifestyle and reduction in body weight [12]. It was reported that a mere 5% weight loss can play a significant role in improvement of PCOS symptoms. In addition, contraceptives such as progesterone tablets or combined contraceptive pill can be used to promote regular menstrual cycle. Further, fertility issues are also addressed using medications such as clomiphene, and off-label use of letrozole and metformin in women intending to conceive [12].

Pakistan has an infertility rate of 21.9% and out of this, 38.5% of infertility is attributable to PCOS [13]. Besides, studies conducted in Pakistani women report a high prevalence for PCOS [4, 14]. A study conducted among women with PCOS visiting clinics in three cities of Pakistan reported that most of them had obesity, hirsutism, elevated blood glucose, irregular menstrual cycles, and acne. The study also reported a low quality of life and depression among respondents [4]. In another study, it was reported that among women with PCOS, sexual dysfunction was a predictor of depression [14].

It has been reported that increasing awareness about the disease led to better management of negative outcomes associated with PCOS. Colwell and colleagues reported that following a clinical research study, women with PCOS felt empowered regarding disease knowledge and were motivated to follow preventive strategies [9]. A study in Saudi Arabia highlighted the increase in knowledge of participants after participating in an awareness initiative through social media [15]. There is a dearth of literature in the area of disease awareness, risks assessment, and perceptions towards PCOS among Pakistani women. A few studies have reported that most Pakistani women lacked adequate awareness about disease and are at an increased risk of having PCOS [16].

Better awareness about PCOS among women could significantly improve understanding of one’s condition. This could open channels for treatment at an early age that would have increased chances of success [8]. Such a study has never been conducted in Pakistani undergraduate students. Hence, this presents an excellent opportunity to evaluate the knowledge, attitude, and perceptions of Pakistani undergraduate students towards PCOS. This may provide a better understanding of the underlying determinants behind a high prevalence of the disease in this population. Hence, the aim of the study was to evaluate knowledge, attitude, perceptions, and assess the determinants of polycystic ovarian syndrome (PCOS) among undergraduate students.

## Methods

### Design, duration, and study venues

A cross-sectional study was conducted for 5 months among female undergraduate students studying in Pakistani academic institutions. The venues of the study were the Dow University of Health Sciences in Karachi and Islam Medical and Dental College in Sialkot, Pakistan. In addition, the survey was also sent to other students online via social media platforms namely WhatsApp ® and Facebook ®.

### Participants’ eligibility criteria

Female undergraduate students aged 18 years and above were invited to participate in the study. Female students with pregnancy, planned pregnancy, and clinical complications of reproductive system were not included considering the nature of the study. Incomplete survey forms were not included.

### Sampling strategy and sample size calculation

Convenience sampling was conducted to gather a large number of responses. According to the Higher Education Commission of Pakistan, the number of female undergraduates enrolled in Pakistani universities and constituent colleges was 585,000 in the year 2014–15 [17]. These figures were latest available figures to date and were considered as the total number of female undergraduate students in Pakistani academia for the purpose of sample size calculation. The sample size was calculated using the following formula [18].

n=N/(1+Nd^2)

Where n = required sample size, N = Population size and d = margin of error (ideal value is 5%) for 95% confidence level. Based on the formula the required sample was 399.73. The sample size after accounting for potential non-response rate was calculated using the following formula [19].

n_n=n/(1‐d)

Where, n_n = sample size after accounting for the non-response rate, n = calculated sample size and d = non-response rate. Thus, n = 394.75 and d = 30%. The final sample required for the study was 566.76. We managed to record responses from 656 samples in the study and analyzed 646 after excluding 10 incomplete responses.

### Research instrument

The study used a specially designed and validated questionnaire. The questionnaire was prepared by a review of literature and through an expert consensus. The panel of experts consisted of 8 academic researchers. The members of the panel included 6 pharmacists, a statistician, and a medical practitioner by profession. It was validated through content and face validation. Before the actual study, the questionnaire was pretested in 9 participants for its suitability. No difficulty in language comprehension and meaning was reported. The questionnaire was developed and validated in English language as it is the official language of instruction in most undergraduate programs offered in Pakistani universities.

The questionnaire consisted of several section. The section 1 contained demographic information. The demographic section included items such as age, college, university, year of study, marital status, monthly family income, height, weight, disease profile, use of contraceptives, duration of menstrual periods, having PCOS, family history of PCOS, history of pregnancy and, source of information regarding PCOS. All of the questions in the demographic sections were developed by an expert consensus. Besides, section 2 contained questions related to the PCOS disease knowledge. It consisted of 20 questions adopted from the study by Colwell et al. [9] We added 3 options, namely, yes, no, and unsure, to each question. The correct answers were awarded a score of one (1). A minimum score of zero (0) while a maximum score of twenty (20) was possible. A higher score represented more correct answers. While lower score showed that the respondents answered incorrectly. A higher score indicated better knowledge. The scores were continuous in nature and no cut-off was defined. Permission was taken from the developers.

In addition, section 3 contained the Ferriman-Gallwey Scale for quantifying hirsutism [20]. The scale contains pictorial representation of hair growth in 9 areas of the body. Each area is shown by pictures indicating some level of hair growth with incremental numbering. Participants can select the picture that is most relevant to their condition, they can also decide if it is not applicable in case there is no hair growth. The total score ranges from 0 to 36 and a cut-off of 8 is considered to identify hirsutism. Less than 8 denotes normal hair growth. Several categories are defined, that is, a score between 8 to 15 is categorized as mild hirsutism while score greater than 15 shows moderate/severe hirsutism [20]. Further, section 4 consisted of a clinical risk assessment questionnaire adopted from the work of Pedersen and colleagues [21]. It contained 4 questions namely duration of menstrual period, hair growth during menstrual cycle, obesity status, and discharge from breast. The last section, section 5 documented their beliefs about PCOS in Pakistan, perceptions about clinical features of PCOS, reason for undergoing screening, feelings about screening, societal pressures, preference of gynecologists in terms of gender, barriers towards screening, etc.

### Data collection process

The data were collected using an online link as well paper-based survey questionnaire. The survey was created on an online survey platform and was distributed to students via social media platforms. The hardcopy of the survey was provided to the students in their free time (self-study time) at the campus by some of the investigators. They had the option of returning the survey at a later date. A drop-box was kept at the office of investigator to collect the survey forms from students. The survey was anonymous and no personal information was recorded. The online link was distributed using social media platforms to the students. The students were advised to fill the response once either online or through physical survey.

### Study outcomes

The study outcome identified were the disease knowledge, and perceptions and attitude towards PCOS and, the determinants of having PCOS. The secondary outcomes were the self-reported occurrence/magnitude of PCOS, contraceptive use, and familial history of PCOS. The secondary outcomes were calculated using an online calculator [22]. The positive responses of participants were considered as ‘True positive’ while the negative responses were entered as ‘True negative’. The responses of participants who indicated ‘Maybe’, were entered at two places, that is, one time as ‘False positive’ and second time as ‘False negative’ in the calculator.

### Data management and presentation

The data were analyzed using IBM SPSS version 23. The categorical data such as demographic information, BMI categories, hair growth categories, etc., were presented using sample counts (N) and frequencies (%). The continuous data such as age, knowledge score, hirsutism score, BMI, etc., were reported using descriptive statistics such as mean ($\bar{x}$) and standard deviation (SD), or median ($\bar{x}$) and interquartile range (IQR), depending upon data distribution. Range (R) was also used in reporting continuous data. Inferential statistics namely the simple and multiple regression analysis were conducted to uncover the determinants of PCOS knowledge and self-reported occurrence. Linear regression was conducted to analyze the determinants of PCOS knowledge score as it was discrete data while logistic regression was used to report the determinants of having PCOS since it was a categorical dichotomous variable. Analyses for model fit were also conducted as a part of regression analyses and the results are presented at footnotes in respective Tables 3 and 4.

### Participant consent and ethical clearance

The participants were briefed about the study and were asked to provide their consent. The nature of consent was implied, that is, without personal identifiers. Those who consented to participate were provided with the questionnaire. Participants were told that their decision to participate would not affect the education and related services that they were entitled to. They were also informed that their response was been recorded without any personal identifiers and therefore, it would not be possible to withdraw their response once it was submitted. The study was approved by the Institutional Review Board of Dow University of Health Sciences in Karachi (IRB-2087/DUHS/Approval/2021/438) and the Ethical Review Board of Islam Medical and Dental College in Sialkot (2022-05/PhP), Pakistan.

## Results

A total of 646 responses were gathered. The average age of participants was 21.74 ± 1.73 years. Most participants were single (N = 595, 92.1%), studied in medical college (N = 540, 83.6%), studied in public sector universities (N = 369, 57.1%), and had a monthly family income between PKR 50,000–100,000, that is, USD 243.54–487.09 (N = 328, 50.8%). The majority of responses were from students in their final year of study (N = 256, 39.6%) (Table 1).

**Table 1:** Background characteristics of participants.

Characteristics	Frequency (N)	Percent (%)
College		
Non-Medical	106	16.4
Medical	540	83.6
University		
Private	277	42.9
Public	369	57.1
Year of Study		
1^st^ Year	72	11.1
2^nd^ Year	125	19.3
3^rd^ Year	26	4.0
4^th^ Year	167	25.9
5^th^ Year	256	39.6
Marital Status		
Married	51	7.9
Single	595	92.1
Family monthly Income (PKR)*		
Between PKR 50,000–100,000 (USD 243.54–487.09)	328	50.8
Less than PKR 50,000 (< USD 243.54)	177	27.4
More than PKR 100,000 (> USD 487.09)	141	21.8

*1 USD equals 205.31 Pakistani Rupee

The average body mass index (BMI) of students was 21.81 ± 4.52 kg/m^2^. More than half of the sample had a normal BMI (N = 371, 57.4%). Of those who were obese (N = 24, 3.8%), 14 were categorized as obese class I, and 5 each fell under obese class II and III. A small number of participants had PCOS (N = 62, 9.6%), most participants have never been pregnant (N = 622, 96.3%), and did not have anyone in family with PCOS (N = 500, 77.4%). Besides, most respondents had no illnesses (N = 540, 83.6%), had regular menstrual periods (N = 432, 66.9%), and never used a contraceptive (N = 599, 92.7%). The notable illnesses reported by students were thyroid disorders, hypertension, asthma, gastrointestinal tract disturbance, diabetes, diabetes with hypertension, ulcer, insomnia, musculoskeletal conditions, migraine, anxiety, epilepsy, depression, anemia, kidney and gall stones, allergies, cancer, and cholecystitis. The median use of contraceptives in a month was 3, (IQR = 9, Range = 1–36). The average duration of menstrual period was reported at 5 ± 1.68 (Range 2–9) (Table 2).

**Table 2:** Medical information of participants.

Characteristics	Frequency (N)	Percent (%)
BMI category		
Underweight	148	22.9
Normal range	371	57.4
Overweight	103	15.9
Obese	24	3.8
Illnesses		
No	540	83.6
Yes	106	16.4
Use of contraceptives		
Maybe	26	4.0
No	599	92.7
Yes	21	3.3
Self-description of menstrual periods		
I am not sure	68	10.5
Irregular	146	22.6
Regular	432	66.9
Have Polycystic Ovarian Syndrome (PCOS)?		
Maybe	69	10.7
No	515	79.7
Yes	62	9.6
Anyone in family for instance, mother, or sister have PCOS?		
Maybe	52	8.0
No	500	77.4
Yes	94	14.6
History of pregnancy		
Attempted to become pregnant with successful conception and delivery of baby	18	2.8
Attempted to become pregnant without success for more than 1 year	6	0.9
I have never been pregnant	622	96.3

### Primary outcomes

The average PCOS knowledge score was 11.5 ($\bar{x}$ = 11.58, SD = 4.99, R = 0–20). The average PCOS knowledge score was 12 ($\bar{x}$ = 12.02, SD = 4.73, R = 0–20) for students studying in medical colleges. The average PCOS knowledge score was 9 ($\bar{x}$ = 9.36, SD = 5.65, R = 0–20) for students studying in non-medical colleges. The value for Cronbach’s alpha (α) was 0.861 and 0.863 based on standardized items.

The model for good knowledge of PCOS revealed that the score for PCOS rose by 0.184 for participants who studied in medical college (p<0.001). It was also highlighted that the score for PCOS knowledge increased by 0.094 for participants who studied in a public sector university (p<0.05). Besides, the score increased by 0.125 for students with a menstrual period < 5 days (p<0.01). Also, the score rose by 0.121 for participants with a family member with PCOS (p = 0.001). Some variables such as increasing age, studying in years final years of the degree program, body hair growth, were significant determinants of a higher score for knowledge in univariate analysis. Similarly, a variable of having > 5 days duration of menstrual period was a significant determinant of decreased score in univariate analysis. However, all of these became non-significant when they were adjusted for participants’ demographics (Table 3).

**Table 3:** Predictors of good knowledge of PCOS.

Characteristics	Simple Regression		Multiple Regression		
	**Coefficient (β)**	**p-value**	**Coefficient (β)**	**p-value**	**VIF**
Age (in year)	0.119	0.002	0.059	0.222	1.78
BMI (kg/m^2^)	0.014	0.731	---	---	---
College					
Non-Medical (R)	---	---	---	---	---
Medical	0.197	<0.001	0.184	<0.001	1.04
University					
Private (R)	---	---	---	---	---
Public	0.085	0.031	0.094	0.011	1.03
Year of Study					
≤ 3^rd^ Year (R)	---	---	---	---	---
4^th^ and 5^th^ Year	0.141	<0.001	0.058	0.231	1.81
Marital Status					
Married (R)	---	---	---	---	---
Single	0.026	0.505	---	---	---
Family monthly income in PKR (USD)*					
> PKR 100,000, (> USD 487.09) (R)	---	---	---	---	---
≤ PKR 100,000, (≤ USD 487.09)	0.060	0.125			
Illnesses					
No (R)	---	---	---	---	---
Yes	-0.015	0.702	---	---	---
Use of contraceptives					
No (R)	---	---	---	---	---
Yes	0.016	0.682	---	---	---
Duration of menstrual period					
Other (R)	---	---	---	---	---
< 5 days	0.118	0.003	0.125	0.004	1.42
Duration of menstrual period					
Other (R)	---	---	---	---	---
> 5 days	-0.083	0.035	-0.010	0.809	1.41
Self-description of menstrual periods					
Regular (R)	---	---	---	---	---
Irregular	0.032	0.424	---	---	---
Anyone in family, i.e., mother, or sister with PCOS?					
No (R)	---	---	---	---	---
Yes	0.122	0.002	0.121	0.001	1.01
Pregnancy status					
No (R)	---	---	---	---	---
Yes	-0.058	0.144	---	---	---
Ever sought information about PCOS?					
No (R)	---	---	---	---	---
Yes	0.297	<0.001	0.271	<0.001	1.02
Body hair growth†					
Normal (R)	---	---	---	---	---
Hirsutism	0.001	0.973	---	---	---

†Ferriman-Galway scale for hirsutism

*1 USD equals 205.31 Pakistani Rupee

Multiple regression model applied. Model fitness tested by: ANOVA (F = 16.365, p <0.001); R^2^ = 0.170 and adjusted R^2^ = 0.160

Most of the students mentioned irregular menstrual periods as a clinical feature of PCOS (N = 263, 40.7%) and mentioned the same as a reason to undergo screening if needed in future (N = 307, 47.5%). Slightly less than half of students felt that it would be better to undergo screening (N = 315, 48.8%). More than half of the sample did not feel embarrassed talking about PCOS in the society (N = 443, 68.6%), never discussed it with their doctor (N = 435, 67.3%), and preferred female gynecologist for consultation (N = 508, 78.6%). A third of participants highlighted the lack of self-knowledge as a barrier towards PCOS screening (N = 204, 31.6%) followed by slightly less than a quarter of students highlighting shyness/ reluctance (N = 138, 21.4%). The majority of students acknowledged it to be a major health issue in Pakistan (N = 504, 78%). The severity of PCOS for Pakistani women was checked on a scale of 1–10 where 1 meant no severity and 10 meant extreme severity was used. The severity of PCOS was rated between 7–8 ($\bar{x}$ = 7.75, SD = 1.76, R = 1–10). The majority of students tried to seek information about PCOS in the past (N = 433, 67%) and used multiple sources (N = 194, 29.8%). The details are presented in Table 4.

**Table 4:** Attitude and perceptions towards PCOS.

Variables	Frequency	Percent
Participants’ opinion of a clinical feature of PCOS		
Cysts on ovaries	156	24.1
Excess hair growth	16	2.5
Hormone imbalance/ high blood androgen levels/ excess male hormones	149	23.1
Increased risk of metabolic complications (type 2 diabetes/gestational diabetes/ cardiovascular disease)	12	1.9
Increased tendency for weight gain	19	2.9
Insulin resistance	7	1.1
Irregular menstrual cycles/periods	263	40.7
Mood disorders (depression and anxiety)	12	1.9
Reduced fertility or infertility	12	1.9
Reason for undergoing screening for PCOS if ever needed		
Advice of friend, husband, etc.	12	1.9
Doctor’s advice	104	16.1
Family member having PCOS	49	7.6
Fear of having the disease	127	19.7
Irregular periods	307	47.5
Media awareness	47	7.3
Feelings about undergoing screening for PCOS at hospital or clinic		
I have religious issues in doing so	9	1.4
It is culturally unacceptable	14	2.2
It is expensive	22	3.4
Only when the need arises	286	44.3
Yes, it is better	315	48.8
Feel embarrassed about talking about PCOS in the society?		
Maybe	119	18.4
No	443	68.6
Yes	84	13.0
Ever discussed this disease with doctor?		
Maybe	26	4.0
No	435	67.3
Yes	185	28.6
Preference of gynecologist for a consultation in terms of gender		
Anyone	131	20.3
Female	508	78.6
Male	7	1.1
Barriers to undergo PCOS screening		
Culture / traditions	48	7.4
Fear of diagnosing the disease	116	18.0
Lack of self-knowledge	204	31.6
No facilities	59	9.1
No female doctor/ Not to be examined by male doctor	61	9.4
Shyness / Reluctance	138	21.4
Societal pressure	20	3.1
Do you think it is becoming a major problem of Pakistani women?		
Maybe	125	19.3
No	17	2.6
Yes	504	78.0
Ever sought information about PCOS?		
No	213	33.0
Yes	433	67.0
Source of information (N = 650)		
Health literature (printed /electronic) Health literature (printed /electronic)	110	16.9
Courses related to studies	80	12.3
Physician/doctor	55	8.5
Not applicable / I never sought information about PCOS	96	14.8
Other sources	61	9.4
Family/relatives	54	8.3
More than one source	194	29.8

### Secondary outcomes

The occurrence of self-reported PCOS was 18.32% (95% CI: 15.55% to 21.36%). The occurrence for self-reported PCOS in family was 20.92% (95% CI: 17.96 to 24.13%). The prevalence of self-reported contraceptives use was 6.99% (95% CI: 5.18% to 9.19%). The average Ferriman-Gallwey score for hirsutism was 14 (X = 14.16, SD = 6.18, R = 7–36). The value for Cronbach’s alpha (α) was 0.893 and 0.894 based on standardized items. Most participants had mild hirsutism (N = 283, 43.8%). A third had moderate/severe hirsutism (N = 225, 34.8%). Less than a quarter of the sample had normal hair growth (N = 138, 21.4%).

### Predictors of having PCOS

The model of PCOS revealed that overweight (AOR = 1.79, p<0.05) and obese participants (AOR = 2.34, p<0.05) had higher likelihood of having PCOS as compared to the others with normal BMI. Besides, participants who indicated that they used contraceptives were 4 times more likely to have PCOS (AOR = 4.12, p<0.01) as compared to those who never used them. In addition, participants who described their menstrual periods as being irregular were 3 to 4 times more likely to have PCOS (AOR = 3.7, p<0.001). Also, those who had a family member with PCOS were twice more likely to have PCOS (AOR = 2.01, p<0.05). Students who had a higher score for hirsutism, i.e., moderate/severe hirsutism, were 4 times more likely to have PCOS (AOR = 4.44, p<0.05).

Other demographic factors such as participants with a monthly family income less than PKR 50,000 were 2 times more likely to have PCOS (AOR = 2.31, p<0.05). Some variables such as having illnesses, and studying in a non-medical college, appeared significant determinants in univariate analysis but became non-significant when they were adjusted for participants’ demographic variables (Table 5).

**Table 5:** Predictors of having PCOS.

Characteristics	OR (95% CI of OR)	AOR (95% CI of OR)
Age (in year)	0.91 (0.79, 1.06)	---
College		
Medical (R)	---	---
Non-Medical	2.10 (1.15, 3.84)*	1.61 (0.78, 3.31)
University		
Private (R)	---	---
Public	1.12 (0.66, 1.92)	---
Year of Study		
4^th^ and 5^th^ Year (R)	---	---
≤ 3^rd^ Year	1.42 (0.83, 2.42)	---
Marital Status		
Single (R)	---	---
Married	1.86 (0.83, 4.17)	---
Family monthly Income in PKR (USD)*		
PKR 50,000–100,000, (USD 243.54–487.09) (R)	---	---
< PKR 50,000, (< USD 243.54)	2.65 (1.43, 4.90)*	2.31 (1.13, 4.71)*
> PKR 100,000, (> USD 487.09)	1.97 (1.09, 3.93)*	1.47 (0.66, 3.26)
BMI category		
Normal range (R)	---	---
Underweight	0.41 (0.17, 1.09)	0.45 (0.16, 1.26)
Overweight	3.20 (1.75, 5.86)***	1.79 (1.09, 3.67)*
Obese	3.93 (1.45, 10.67)**	2.34 (1.27, 8.20)*
Illnesses		
No (R)	---	---
Yes	2.10 (1.15, 3.84)*	1.16 (0.55, 2.44)
Use of contraceptives		
No (R)	---	---
Yes	6.98 (3.58, 13.61)***	4.12 (1.82, 9.59)**
Duration of menstrual period		
Equal to 5 days (R)	---	---
Less than 5 days	2.54 (1.23, 5.27)*	1.65 (0.71, 3.85)
More than 5 days	1.36 (0.67, 2.77)	1.12 (0.49, 2.54)
Self-description of menstrual periods		
Regular (R)	---	---
Irregular	6.50 (3.62, 11.68)***	3.70 (1.94, 7.05)***
Anyone in family, i.e., mother, or sister with PCOS?		
No (R)	---	---
Yes	2.49 (1.36, 4.57)**	2.01 (1.19, 4.18)*
Pregnancy status		
No (R)	---	---
Yes	1.95 (0.64, 5.88)	---
Ever sought information about PCOS?		
No (R)	---	---
Yes	8.08 (2.89, 22.57)***	6.30 (2.14, 18.59)**
Body hair growth		
Normal (R)	---	---
Mild	4.36 (1.29, 14.70)*	2.75 (0.76, 9.94)
Moderate/Severe	8.01 (2.41, 26.62)**	4.44 (1.24, 15.91)*

*1 USD equals 205.31 Pakistani Rupee

R = reference case, OR = Odds ratio, CI = Confidence Interval; AOR = Adjusted odds ratio * = P<0.05

** = P<0.01, and

*** = P<0.001. Multiple logistic regression, ‘Enter’ method was applied; Multicollinearity were checked and not found; Hosmer-Lemeshow test, (χ2 = 5.44, p = 0.710); Pearson chi-square and Significant for Model (χ2 = 118.18 and p< 0.001) and Classification table (overall correctly classified percentage = 90.9) were applied to check the model fitness. Cox & Snell R Square = 0.167; Nagelkerke R Square = 0.357.

## Discussion

This was a multi-center study that was conducted to assess the risk of having PCOS, knowledge about the disease and perceptions of undergraduate female students about PCOS. The demographic profile reflects true enrollment status of undergraduate female students in Pakistani academic settings as most of students are in the same age group and are single. However, most of our sample was from medical college. This may be due to the nature of the study as females from non-medical background might not have been convinced to attempt the survey.

The findings highlight that the average disease knowledge score was reported to be 11.5/20 that accounts for 57.5% of correct answers. This finding showed that the knowledge about the disease was inadequate among participants. Besides, participants who studied in the medical college had a slightly better score, that is, 12 which accounted for 60% correct responses. However, the score was 9 for participants hailing from non-medical colleges. This was even lower than 50%. In this context, a study by Tahir and colleagues reported that almost three quarters of participants from medical colleges were aware of the disease [23]. The study did not use a standardized and pre-tested knowledge scale to document the knowledge score of participants and was conducted in students from medical and dental colleges. We used a standardized questionnaire to report knowledge score and our sample included participants from medical and non-medical backgrounds. These aspects could be regarded as strength of our study.

Based on the regression analysis it was found that the score for knowledge increased if the participants were studying in medical college. It also increased if the students had a family history of PCOS. The former occurrence is related to the nature of education. It is logical as medical curriculum is designed to include information about diseases that would include PCOS as well. However, the latter is due to the experience of living with the disease. Therefore, having someone in family with PCOS would have exposed the students to it and consequently, students would have come across the nature of disease, signs and symptoms, and its management. Hence, they appeared more knowledgeable. Though, it is an assumption and not based on evidence.

Most of the students mentioned irregular menstrual periods as a clinical feature of PCOS however, some participants also mentioned cardiovascular, endocrine, and mental complications such as depression and anxiety. It is pertinent to mention here that PCOS is linked to several complications that include the above-mentioned diseases as well [4, 24]. Most students mentioned the same as a likely reason to undergo screening if needed in future. Slightly less than half of students felt that it would be better to undergo screening. More than half of the sample did not feel embarrassed talking about PCOS in the society. However, they never discussed it with their doctor, and preferred female gynecologist for consultation. This highlights that although the topic is not regarded as a taboo, there is still a sense of hesitancy to discuss the same.

A third of participants highlighted the lack of knowledge as a barrier towards PCOS screening followed by slightly less than a quarter of students highlighting shyness/reluctance. This occurrence indicates acknowledgement of the problem among the participants. It is positive as acknowledging a problem is the first step towards addressing it. Therefore, it requires concerted efforts to address the knowledge debacle. Considering the example of universities in Saudi Arabia to increase awareness of women specific disease such as breast cancer [25], universities in Pakistan can follow suit and observe a month of awareness of PCOS, conduct awareness campaigns during this time, distribute educational materials and pamphlets, merchandize with awareness logo and signage, etc., and arrange talks or seminars for their students with renowned professionals having substantial contribution in this field.

This exercise would certainly improve the level of understanding and knowledge. Moreover, having free distribution of merchandise such as keychains, shirts, stationery items, etc., with appropriate signage would communicate the intended message non-verbally, and promote a healthy discussion about the disease openly in the society. This has the potential to further lower down the levels of hesitancy and stigma toward its discussion. The majority of students recognized PCOS as a major health issue in Pakistan and rated the severity of PCOS between 7–8 on a scale of 10. This shows that younger lot of women of reproductive age in Pakistan duly recognize the severity of the disease.

Some other notable findings were that the magnitude of self-reported PCOS was 18.32% while the occurrence of PCOS in the family was almost 21%. Studies have highlighted that women in Pakistan have one of the highest prevalence of PCOS in the world [4]. This study found that obesity and family history increased the likelihood of PCOS by 2 times. Besides, participants with irregular menstrual periods were 3–4 times more likely to have PCOS. In addition, most of study sample had some level of hirsutism and those who had moderate to severe hirsutism were 4 times more likely to have PCOS. To this end, a study conducted by Anjum and colleagues among women with PCOS in clinic settings of Pakistan reported hirsutism and oligomenorrhea as the most common features [26]. Further, Anjum et al. also reported that more than 80% of the women with PCOS were obese [26]. In another study conducted among Pakistani women of slightly closer age range to our study sample, it was found that more than 91% of women had a high waist-to-hip ratio (WHR), and a mean value for WHR was reported at 0.88 [27]. According to the WHO, a cut-off value of 0.8 for WHR is recommended for women [28]. Moreover, study by Nazir and colleagues reported similar clinical features [29]. Hence, our findings are in line with these studies [26, 27, 29].

Hirsutism is another common feature in PCOS. Our study reported that there was some level of hirsutism in more than three quarter of our study sample, that is, more than 78%. It is reported that the prevalence of hirsutism in PCOS could be between 70–80% as compared to prevalence of 4–11% for the same among women without PCOS [30]. Hirsutism in Pakistani women has been investigated previously by Malik and colleagues [31]. The study found out that amongst most Pakistani women, the common causes of hirsutism were idiopathic and PCOS [31]. Further, having a positive family is also reported to be a risk factor for developing PCOS. Studies highlight that the risk for developing PCOS is increased by 35% - 40% if mother or sibling has it [32, 33]. This occurrence also highlights the need to conduct large scale studies to evaluate the family history as a risk factor for developing PCOS in Pakistani women.

This study also noted a figure of 6.99% for self-reported contraceptives use. It was revealed in the regression analysis that participants who used contraceptives were 4 times more likely to have PCOS. It is worthwhile mentioning that contraceptives are also used to treat hirsutism [34, 35], apart from their use in treatment of PCOS [35]. This finding may have resulted either due to treatment of hirsutism since three quarter of our sample had some level of hirsutism and/or, as a treatment of PCOS. Therefore, it is not clear and must be interpreted with caution. However, it does highlight the need to review the cause of hirsutism among Pakistani women and rule out PCOS.

This study is not without limitations. The convenience nature of the sampling limits generalizability of findings and had the potential to introduce selection bias. Secondly, the study banks upon the core finding of having PCOS solely on the response given by the participants. It is self-reported and there is a likelihood that some participants may not have been aware or may have had undiagnosed PCOS. Therefore, the results pertaining to the occurrence of PCOS and regression model may have some degree of variability. To address this issue, the result is presented in 95% confidence range while the predictors having statistical significance in multivariate analysis are considered. The actual numbers may unknown and may be higher.

At the same time, the study has several strengths. It is a novel addition to the body of literature pertaining to PCOS in Pakistani women. It uncovers the underlying determinants of knowledge and risk of PCOS. This study also highlights that PCOS is not a taboo in Pakistani society as with some other women health issues however, there is a hesitancy to visit gynecologists among women for the same. These findings may help in formulating some recommendations aimed to improve symptoms attribution.

## Conclusion

The level of knowledge about PCOS in our sample of undergraduate students was found to be inadequate. In addition, medical students do not have the required level of understanding about the disease which is concerning. Lifestyle changes are recommended in this population as obesity was observed to be a determinant of having PCOS. Additionally, females with a familial history of PCOS and hirsutism were observed to have a higher likelihood of PCOS in our study. Therefore, it is recommended to have clinical consultations with gynecologists to rule out PCOS at an early stage. However, there still exist some level of hesitancy to have a discussion with a gynecologist about it, as observed in this study.

The positive aspect is that the topic of PCOS is not considered a taboo in society, there is an acknowledgement of PCOS as a problem for Pakistani women, and lack of self-knowledge about the same, These occurrences in our study show a positive mindset of the society towards addressing this health issue. Observing an awareness month within academic premises, conducting campaigns, promoting disease education, distributing merchandise with disease awareness logo and signage, would greatly help in raising awareness of the disorder and lowering stigma and hesitancy.

## Supporting information

S1 File Occurrence of contraceptives use. (PDF)

S2 File Occurrence of PCOS in family. (PDF)

S3 File Occurrence of PCOS. (PDF)

S1 Data SPSS raw data. (SAV)
