# Correction: Knowledge, attitude, and perceptions about polycystic ovarian syndrome, and its determinants among Pakistani undergraduate students

**DOI:** 10.1371/journal.pone.0318390

**Published:** 2025-01-24

**Authors:** The PLOS ONE Staff

## Notice of republication

This article was republished on 16^th^ January 2025, to correct an error in PDF. Tables 1–5 were missing from the article PDF. The publisher apologises for this error.

## Supporting information

S1 File Originally published, uncorrected article. (PDF)

S2 File Republished, corrected article. (PDF)
