# Supplementary material for: Knowledge, attitude, and perceptions about polycystic ovarian syndrome, and its determinants among Pakistani undergraduate students
Source: PLoS One. 2023 May 25;18(5):e0285284. doi: 10.1371/journal.pone.0285284 (PMC10212137; doi:10.1371/journal.pone.0285284)
Supplement: S2 File — (PDF) [file pone.0285284.s002.pdf]

MedCalc®
easy-to-use statistical software

Free statistical calculators

Diagnostic test evaluation calculator

Instructions: enter the number of cases in the diseased group that test positive (*a*) and negative (*b*); and the number of cases in the non-diseased group that test positive (*c*) and negative (*d*).

Disease prevalence

If the sample sizes in the positive (Disease present) and the negative (Disease absent) groups do not reflect the real prevalence of the disease, you can enter the disease prevalence (expressed as a percentage) in the corresponding input box.

Next click the **Test** button.

	**Disease**				
**Test**	**Present**	**n**	**Absent**	**n**	**Total**
**Positive**	True Positive	a= 94	False Positive	c= 52	a + c = **146**
**Negative**	False Negative	b= 52	True Negative	d= 500	b + d = **552**
**Total**		a + b = **146**		c + d = **552**	

Disease prevalence

If the ratio of cases in the Disease Present and Disease Absent groups does not reflect the disease prevalence, enter:

disease prevalence (%):

Test

Results

**Statistic**	**Value**	**95% CI**
Sensitivity	64.38%	56.04% to 72.13%
Specificity	90.58%	87.83% to 92.88%
Positive Likelihood Ratio	6.83	5.14 to 9.09
Negative Likelihood Ratio	0.39	0.32 to 0.49
Disease prevalence (*)	20.92%	17.96% to 24.13%

Positive Predictive Value (*)	64.38%	57.61% to 70.63%
Negative Predictive Value (*)	90.58%	88.53% to 92.29%
Accuracy (*)	85.10%	82.24% to 87.66%

(*) These values are dependent on disease prevalence.

Definitions

- *Sensitivity*: probability that a test result will be positive when the disease is present (true positive rate).

  = a / (a+b)

- *Specificity*: probability that a test result will be negative when the disease is not present (true negative rate).

  = d / (c+d)

- *Positive likelihood ratio*: ratio between the probability of a positive test result given the *presence* of the disease and the probability of a positive test result given the *absence* of the disease, i.e.

  = True positive rate / False positive rate = Sensitivity / (1-Specificity)

- *Negative likelihood ratio*: ratio between the probability of a negative test result given the *presence* of the disease and the probability of a negative test result given the *absence* of the disease, i.e.

  = False negative rate / True negative rate = (1-Sensitivity) / Specificity

- *Positive predictive value*: probability that the disease is present when the test is positive.

$$PPV = \frac{sensitivity \times prevalence}{sensitivity \times prevalence + (1 - specificity) \times (1 - prevalence)}$$

- *Negative predictive value*: probability that the disease is not present when the test is negative.

$$NPV = \frac{specificity \times (1 - prevalence)}{(1 - sensitivity) \times prevalence + specificity \times (1 - prevalence)}$$

- *Accuracy*: overall probability that a patient is correctly classified.

  = Sensitivity × Prevalence + Specificity × (1 − Prevalence)

Sensitivity, specificity, disease prevalence, positive and negative predictive value as well as accuracy are expressed as percentages.

Confidence intervals for sensitivity, specificity and accuracy are "exact" Clopper-Pearson confidence intervals.

Confidence intervals for the likelihood ratios are calculated using the "Log method" as given on page 109 of Altman et al. 2000.

Confidence intervals for the predictive values are the standard logit confidence intervals given by Mercaldo et al. 2007.

Literature

- Altman DG, Machin D, Bryant TN, Gardner MJ (Eds) (2000) Statistics with confidence, 2nd ed. BMJ Books.
- Gardner IA, Greiner M (2006) Receiver-operating characteristic curves and likelihood ratios: improvements over traditional methods for the evaluation and application of veterinary clinical pathology tests. Veterinary Clinical Pathology 35:8-17.
- Griner PF, Mayewski RJ, Mushlin AI, Greenland P (1981) Selection and interpretation of diagnostic tests and procedures. Annals of Internal Medicine 94:555-600.
- Hanley JA, McNeil BJ (1982) The meaning and use of the area under a receiver operating characteristic (ROC) curve. Radiology 143:29-36.
- Mercaldo ND, Lau KF, Zhou XH (2007) Confidence intervals for predictive values with an emphasis to case-control studies. Statistics in Medicine 26:2170-2183.
- Metz CE (1978) Basic principles of ROC analysis. Seminars in Nuclear Medicine 8:283-298.
- Zhou XH, NA Obuchowski, DK McClish (2002) Statistical methods in diagnostic medicine. New York: Wiley.
- Zweig MH, Campbell G (1993) Receiver-operating characteristic (ROC) plots: a fundamental evaluation tool in clinical medicine. Clinical Chemistry 39:561-577.

How to cite this page

- MedCalc Software Ltd. Diagnostic test evaluation calculator. https://www.medcalc.org/calc/diagnostic_test.php (Version 20.114; accessed September 7, 2022)

Recommended book

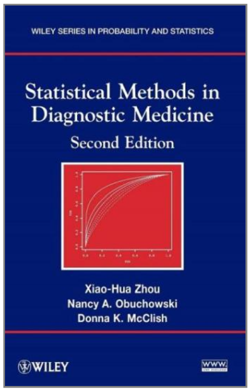

Statistical Methods in Diagnostic Medicine
Xiao-Hua Zhou, Nancy A. Obuchowski, Donna K. McClish

Buy from Amazon

Statistical Methods in Diagnostic Medicine provides a comprehensive approach to the topic, guiding readers through the necessary practices for understanding these studies and generalizing the results to patient populations. Following a basic introduction to measuring test accuracy and study design, the authors successfully define various measures of diagnostic accuracy, describe strategies for designing diagnostic accuracy studies, and present key statistical methods for estimating and comparing test accuracy.

https://www.medcalc.org/calc/diagnostic_test.php

© 2022 MedCalc Software Ltd
